# Diagnosing the frontal variant of Alzheimer’s disease: a clinician’s yellow brick road

**DOI:** 10.1186/s40734-017-0052-4

**Published:** 2017-03-02

**Authors:** Russell P. Sawyer, Federico Rodriguez-Porcel, Matthew Hagen, Rhonna Shatz, Alberto J. Espay

**Affiliations:** 1 Department of Neurology, University of Cincinnati, 260 Stetson Street Suite 2300, Cincinnati, OH 45219 USA; 2 UC Memory Disorders Center, University of Cincinnati, 234 Goodman Street, Cincinnati, OH 45219 USA; 3 Gardner Family Center for Parkinson’s disease and Movement Disorders, University of Cincinnati, 234 Goodman Street, Cincinnati, OH 45219 USA; 4 Department of Pathology and Laboratory Medicine, University of Cincinnati, 234 Goodman Street, Cincinnati, OH 45219-0533 USA

**Keywords:** Frontal variant of Alzheimer’s disease, Frontotemporal Dementia, Movement disorders, Parkinsonism, Behavioral disorder

## Abstract

**Background:**

Disruption of the frontal lobes and its associated networks are a common consequence of neurodegenerative disorders. Given the wide range of cognitive, behavioral and motor processes in which the frontal lobes are involved, there can be a great variety of manifestations depending on the pathology distribution. The most common are the behavioral variant of frontotemporal dementia (bvFTD) and the frontal variant of Alzheimer’s disease (fvAD), which are particularly challenging to disentangle. Recognizing fvAD from bvFTD-related pathologies is a diagnostic challenge and a critical need in the management and counseling of these patients.

**Case presentation:**

Here we present three pathology-proven cases of Alzheimer’s disease initially misdiagnosed as bvFTD and discuss the distinctive or less overlapping historical, examination, and laboratory findings of fvAD and bvFTD, deriving analogies for mnemonic endurance from the Wizard of Oz worldview.

**Conclusion:**

The Yellow Brick Road to diagnosing these disorders may be served by the metaphor of fvAD as the irritable, paranoid, and tremulous Scarecrow and bvFTD the heartless, ritualistic, and rigid Tin Man. An Oz-inspired creative license may help the clinician recognize the differential disease progression, caregiver burden, and treatment response of fvAD compared with bvFTD.

**Electronic supplementary material:**

The online version of this article (doi:10.1186/s40734-017-0052-4) contains supplementary material, which is available to authorized users.

## Introduction

While an amnestic syndrome is the most common presentation of Alzheimer’s disease (AD), atypical variants have been recognized. These include the posterior cortical atrophy syndrome, corticobasal syndrome, logopenic variant of primary progressive aphasia, and frontal variant (fvAD) [1, 2]. FvAD is an under-recognized form of AD, often misdiagnosed as more common frontal lobe syndromes such as the behavioral variant of frontotemporal dementia (bvFTD) or vascular dementia affecting frontal networks. We present three patients with pathology-proven AD presenting with cognitive and behavioral impairment in the context of parkinsonian features, initially diagnosed as bvFTD. We then discuss the features in the history, examination and ancillary testing that may have helped distinguishing these entities. Finally, we suggest analogies from Frank Baum’s novel “The Wonderful Wizard of Oz” to help the reader (without trivializing) remember the phenotypic differences between them.

### CASE #1

**Fig. 1:**
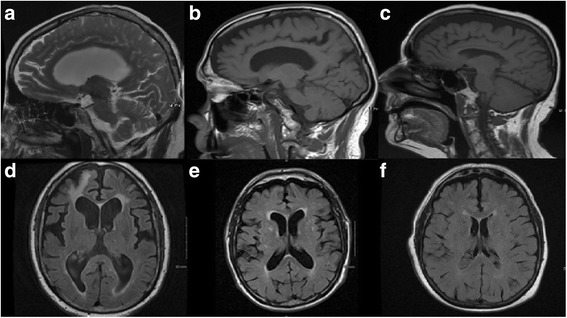
Brain MRI of Case #1–3. Sagittal T2-weighted (*upper row*) and axial FLAIR (lower *row*) brain MRIs. Patient 1 (**a** and **d**) showed mild to moderate atrophy in the frontotemporal regions, minimally asymmetric, with right frontal encephalomalacia. Patient 2 (**b** and **e**) showed similar findings with somewhat lower burden of associated periventricular and spotty subcortical *white* matter increased signal. Patient 3 (**c** and **f**) showed mild diffuse atrophy with minimal periventricular and subcortical *white* matter disease


A 72-year-old man had progressive decline in gait and cognition following a fall 10 months earlier. He was forgetful and his gait shuffling and unstable. In the following months, his family noted a change in personality with apathy and lack of concern for his appearance. His family history was significant for AD in his mother and Parkinson disease (PD) in two paternal cousins. On exam, he was hypophonic and hypomimic. He exhibited symmetric rigidity and bradykinesia and hand myoclonus on outstretched arms. His gait was slow and short-stepped, with stooped posture, reduced arm swing, gait freezing on turns, and inability to tandem walk (Additional file 1: Video S1). Postural reflexes were impaired. Unified Parkinson’s Disease Rating Scale (UPDRS) motor score was 23.5 on initial examination. In addition, he demonstrated bilateral ideomotor apraxia to transitive gestures. Montreal cognitive assessment (MoCA) score was 21/30 with deficits in executive function, phonemic fluency, and attention. Frontal assessment battery (FAB) was 15/18. His brain MRI revealed atrophy in the frontal, temporal, and parietal regions, somewhat worse in the right hemisphere, with increased signal in the periventricular white matter and right frontal lobe (Fig. 1a, d). Patient and family declined amyloid-imaging and cerebral spine fluid (CSF) biomarkers. A diagnosis of possible bvFTD with parkinsonism was made based on apathy, poor personal hygiene, and predominantly executive dysfunction on cognitive testing. Levodopa was titrated to a dose of 1600 mg/day with mild improvement in gait speed but also with development of mild truncal dyskinesia. Clonazepam 0.5 mg daily and venlafaxine 75 mg daily were sequentially added with mild benefit on sleep and depressive symptoms, respectively. His cognition continued to deteriorate despite a trial of rivastigmine. Unfortunately, he later suffered multiple strokes, severely affecting his cognition and ambulation, rendering him wheelchair bound. His condition deteriorated further and he died 7 years after the onset of his symptoms.

Video S1: The video shows the patient’s examination where he exhibits hypophonia and hypomimia. During his motor examination, he evidenced symmetric bradykinesia and distal myoclonus. His gait is slow and short stepped, with stooped posture and reduced arm swing. (MP4 51193 kb)


Brain autopsy showed mild symmetric cerebral atrophy in the frontal and temporal lobes with multiple grossly evident infarcts. The brain weighed 1260 grams. Microscopic evaluation demonstrated moderate to severe neurofibrillary tangles in mesial temporal structures and association areas of the neocortex, Braak stage V and amyloid, Braak, CERAD (ABC) score of B3, associated with frequent neuritic plaques throughout the neocortex, most prominently in the middle frontal, superior and middle temporal gyri, and inferior parietal lobule resulting in C3 scoring on ABC. Along with extensive beta amyloid deposition (A3), these findings were consistent with a high degree of AD neuropathology per the National Institute on Aging-Alzheimer’s Association (NIA-AA) criteria [3–7]. No tau immunoreactive glial deposits were identified. There was mild patchy neuronal loss, moderate neurofibrillary tangles, and scattered senile plaques in the substantia nigra with moderate neuronal cell loss and gliosis in the putamen. There was coexistent cerebrovascular disease, with moderate atherosclerosis of major arteries and diffuse arteriolosclerosis. Remote macroscopic infarcts were noted in the left occipital lobe, right middle frontal gyrus, right anterior medial aspect of superior frontal gyrus, and bilateral cerebellar hemispheres.

### CASE #2

This 79-year-old man presented with a 6-year history of worsening gait and balance. He initially complained of heaviness in his legs followed by forgetfulness and a tendency to stumble and fall. He also manifested word-finding difficulties and impaired visual navigation. Four years after symptom onset, he developed paranoid ideation and anxiety during a trial of levodopa to address presumed PD. Within months, he became more belligerent, disinhibited, irritable and uncharacteristically offensive. He frequently cried and endorsed depression. Word-finding difficulties were compounded by semantic paraphasias (“garage” instead of “cabinet”). He had trouble locating food on his plate when eating. His gait continued to deteriorate with increased freezing and instability, ultimately leaving him wheelchair dependent. His mother developed dementia of unknown etiology in her late seventies. On exam, he was alert but his verbal output truncated with hesitations, semantic and phonemic paraphasias, echolalia and palilalia. He was unable to follow three-step commands. He exhibited hypomimia, bradykinesia and rigidity, but no tremor (Additional file 2: Video S2). UPDRS motor score was 32.5 on initial evaluation. He demonstrated frontal-localizing signs, including grasp reflex and perseverative behaviors. His brain MRI showed moderate atrophy in the frontal and temporal lobes, with increased periventricular and deep white matter signal abnormality (Fig. 1b, e). Mini-mental status exam score was 28/30 and MoCA 23/30, with deficits in executive function and delayed recall. Visuospatial orientation as per clock drawing and figure copying was normal despite complaints of impaired visual navigation possibly suggesting an attentional deficit. A diagnosis of probable bvFTD was made based on disinhibition, loss of empathy (based on pejorative comments), frontal dysfunction, and MRI findings. The patient died within a year from his only visit from a myocardial infarction. Amyloid-imaging and cerebrospinal fluid biomarkers were not performed.


Video S2: The video shows the patient’s hesitant speech with both echo and palilalia. Perseveration is also noted when the patient tried to touch his nose with is finger, something he has been asked to do previously. Inability to follow three step commands is also documented. Finally, bilateral grasp reflex is shown. (WMV 13631 kb)


Gross pathology demonstrated symmetric cortical atrophy in the frontal, anterior, and mesial temporal lobes. Gross brain weight was 1150 g. Microscopy revealed a high degree of AD neuropathologic changes based on NIA-AA guidelines: widespread beta amyloid deposition (A3), neuritic plaques within the neocortex (C3), and neurofibrillary tangles throughout the mesial temporal structures and association areas of the neocortex (Braak stage V and B3) [3–7]. Additionally, alpha-synuclein-positive Lewy bodies were noted in the brainstem (including substantia nigra) and limbic regions, with coexistent mild gliosis in the putamen, suggesting coexistent Lewy body disease (Limbic type, Braak stage IV) [7, 8]. The limited distribution of α-synuclein pathology in cerebral structures suggested that it contributed to the parkinsonian features but was unlikely to have caused the memory and behavioral complaints. No tau-immunoreactive glial deposits or any TDP-43 positive inclusions were identified.

### CASE #3

This 78-year-old woman presented with a 9-month history of worsening mood and behavioral changes associated with language difficulties and parkinsonism. Nine months prior to presentation, family members observed she was overly emotional, crying and laughing often without endorsing sadness or happiness. In the following months, the family noticed an asymmetric rest and action tremor in her right hand and gait shuffling, requiring a walker for ambulation 6 months before presentation. Her speech became dysfluent, with echolalia and palilalia. Forgetfulness worsened and she required assistance in all activities of daily living. When going to the bathroom to wash her hands, she would frequently lower her pants and finish urinating in other rooms. On examination, she had a monotone speech with hypophonia, symmetric bradykinesia and rigidity, mild right-hand rest tremor, and impaired postural reflexes (Additional file 3: Video S3). UPDRS III motor score was 47 on initial evaluation. Root, snout, and glabellar reflexes were present. MoCA score was 8/30 with deficits in all domains, except naming. MRI showed mild diffuse generalized atrophy (Fig. 1c, f). Given her behavioral changes with disinhibition and compulsive behaviors, the patient was diagnosed with possible bvFTD associated with parkinsonism. Levodopa was initiated and titrated up to 500 mg four times daily with moderate benefit on her motor symptoms. Multiple medications were tried over the next few years including valproic acid, lamotrigine, aripiprazole, dextromethorphan/quinidine, and oxazepam with only modest benefit for emotional lability. Citalopram provided the sustained improvement in mood. The patient continued to deteriorate and died 10 years after symptom onset.


Video S3: The video shows the patient’s examination where she is crying, although she does not know why. She exhibits wide-base short-stepped gait with absent arm swing and right hand tremor. (MP4 77086 kb)


Autopsy revealed mild to moderate cortical atrophy, more prominent in the frontal lobes. The brain weighed 1030 grams. The histological hallmarks of Alzheimer disease, abundant neuritic plaques (C3) and neurofibrillary tangles (B3), were identified by beta-amyloid and tau immunohistochemistry. Extensive diffuse deposits of beta-amyloid (A3) were also seen in the cerebrum and cerebellum. By NIA-AA criteria, the findings met criteria for “high level” AD neuropathology [3–7]. Rare, scattered tau-positive neurons were identified in the globus pallidus and putamen but insufficient to be classified as FTD-Tau. Minimal focal neuronal cell loss and gliosis is noted in the substantia nigra with occasional neuromelanin-laden macrophages.

## Review

These three cases highlight the extent to which behavioral abnormalities may overshadow cognitive impairment in fvAD, the lesser known focal variant of AD, leading to the clinical misdiagnosis of bvFTD. Some studies have suggested that fvAD accounts for approximately 2–3% of AD, [1, 2], although this figure is likely higher because AD pathology can be found in up to 25% of patients with a clinical diagnosis of bvFTD [9, 10]. The appropriate clinical distinction between fvAD and bvFTD has implications for prognosis, treatment, disease progression, and caregiver burden [11–15].

The clinical diagnosis of bvFTD is currently based on the presence of behavioral changes antedating cognitive deficits with a sensitivity of 76–95% and specificity of 82–95% [16, 17]. Typically, the presentation is characterized by changes in personality and behavior, most notably apathy, disinhibition, loss of self-awareness, and loss of empathy [11, 18]. A dysexecutive cognitive impairment emerges later in the course of the disease with difficulties in sustaining attention and set shifting. bvFTD is most often caused by frontotemporal lobar degeneration (FTLD) proteinopathies, namely tauopathies (of which microtubule-associated protein tau (MAPT) gene mutations are the most common genetic causes), TDP-43 proteinopathies (of which chromosome 9 open reading frame 72 (*C9orf72*) and progranulin (*GRN*) gene mutations, in order of frequency, are most common), and less frequently, fused in sarcoma (FUS) proteinopathies [19]. In cases of bvFTD-associated parkinsonism, tau is the most common underlying proteinopathy [20].

Unlike bvFTD, the phenotype in fvAD is less well delineated, with heterogenous clinical descriptions from case reports and case series. Nevertheless, an early amnestic phase, with memory dysfunction preceding changes in behavior may be more common in fvAD [21]. Not only is the behavior-cognition sequence different between fvAD and bvFTD, but the nature of the impairments may differ as well.

In addition, vascular pathology may confound the evaluation of frontal lobe syndromes, including fvAD and bvFTD. Small and large vessel vasculopathy can disrupt frontal lobe networks in a manner suggestive of (and potentially in addition to) fvAD and bvFTD. The clinical heterogeneity of the clinical entities and overlap of comorbitities make ascertaining the correct diagnosis a humbling experience, as demonstrated by all three of our cases carrying a diagnosis of bvFTD during life but reclassified as fvAD on autopsy.

### The Yellow brick road

**Fig. 2:**
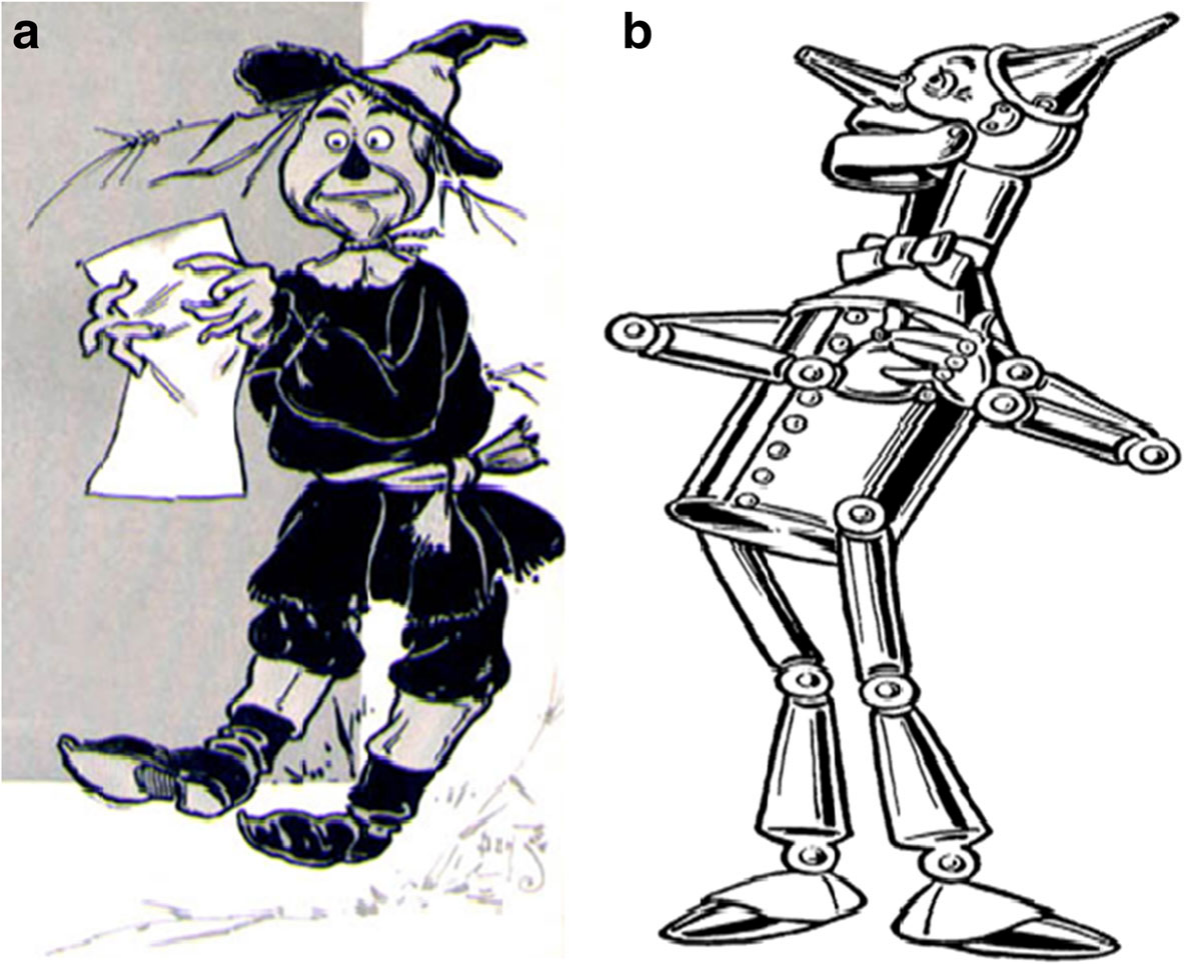
fvAD and bvFTD in the Wizard of Oz. **a** fvAD Scarecrow was searching for a brain because he had none, and without a brain he had no memory or object knowledge. While his phonemic fluency was preserved, fvAD Scarecrow had difficulty with semantics and was irritable and paranoid, believing the crows were gearing to bothering and stealing from him. Furthermore, fvAD Scarecrow was very tremulous (myoclonus). The wind could suddenly jolt him (stimulus-sensitive myoclonus). **b** bvFTD Tin Man had no heart, so his behavior and emotions were affected from the outset. The “heartless” bvFTD Tin Man lacked empathy and was very ritualistic, only going out to chop wood. His rituals included hyperphagia, making him heavier than the straw-filled fvAD Scarecrow. Furthermore, bvFTD Tin Man was insufficiently lubricated, making him appear parkinsonian. This particular bvFTD Tin Man was missing progranulin, rendering his frontotemporal region asymmetric, as judged by a crooked hat. Both images are from 1900; US Copyright law on public domain

Acknowledging the limitations of classic literature-based metaphors to provide a memorable analogy to what an otherwise complex clinical dilemma, we propose considering the “Yellow Brick Road” from the Wizard of Oz characters to remember the clinical variables to consider when facing the fvAD vs. bvFTD clinical distinction (Fig. 2).

The Scarecrow, who had no brain, allegorizes fvAD. Without a brain the fvAD Scarecrow lost the ability to consolidate new memories and object knowledge (Fig. 2a). Although fvAD Scarecrow can produce a list of words based on letters, suggesting an intact phonemic fluency, the meanings of words is lost and therefore semantic fluency and semantic knowledge are impaired. fvAD Scarecrow is *irritable and paranoid*, believing the crows are swarming to torment and steal from him. Furthermore, fvAD Scarecrow is shaky and tremulous, movements that on closer inspection represent myoclonus. The fvAD Scarecrow is also sensitive, the slightest whisper or touch can startle him, because of stimulus-sensitive myoclonus.

The Tin Man symbolizes bvFTD (Fig. 2b). bvFTD Tin Man has no heart, so his behaviors and emotions *lack empathy*. He engages in *ritualistic* and perseverative behaviors, repetitively venturing to the forest to chop wood and gorge. His rituals included hyperphagia, making him heavier than the fvAD Scarecrow. Furthermore, bvFTD Tin Man is insufficiently lubricated: the rust at the joints slow him to a parkinsonian state.

### Chronology of symptoms

**Fig. 3:**
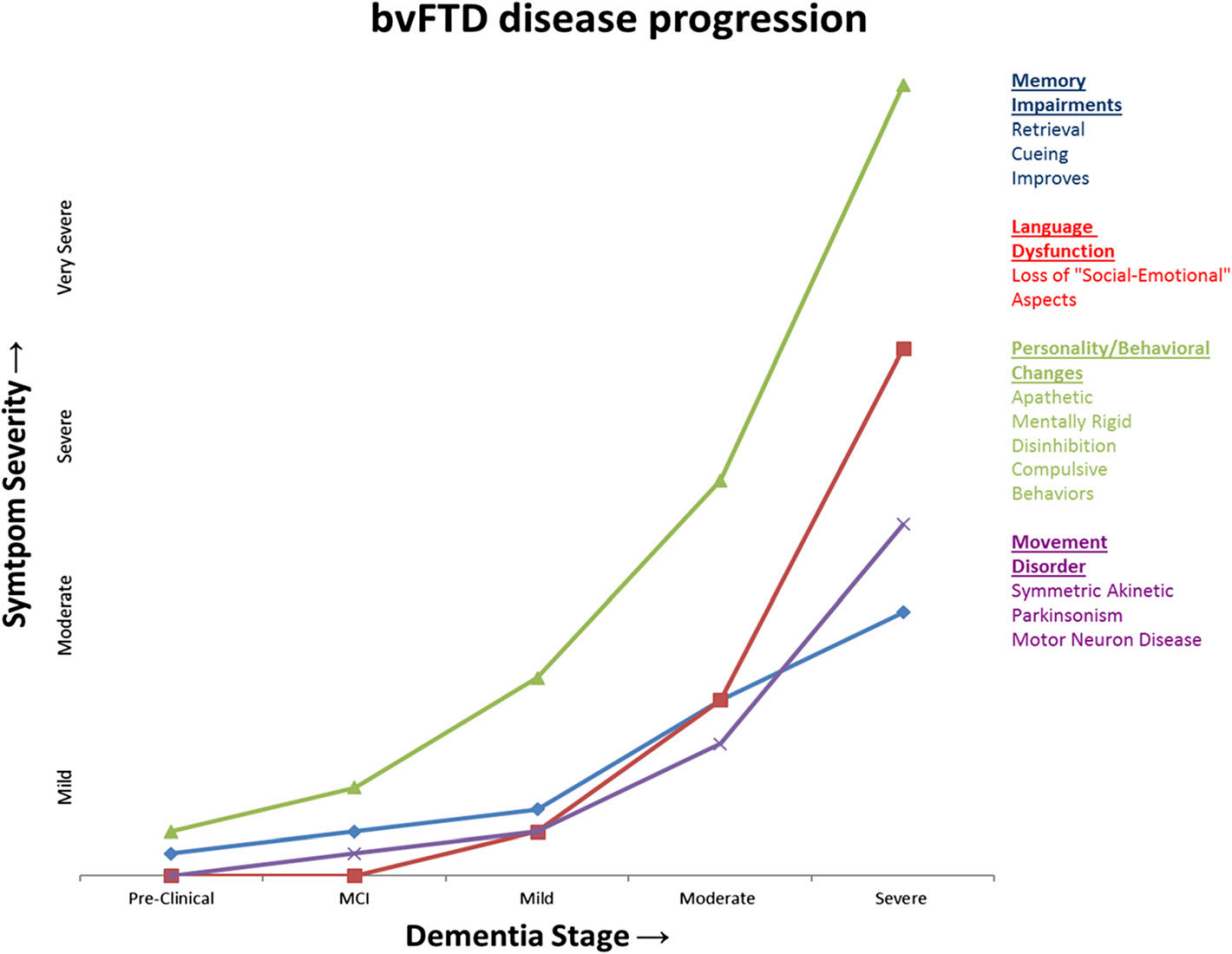
Chronology of symptoms. The diagram illustrates the severity and timing of cognitive impairments, motor manifestations, and behavioral changes as they may appear across disease stages in bvFTD

**Fig. 4:**
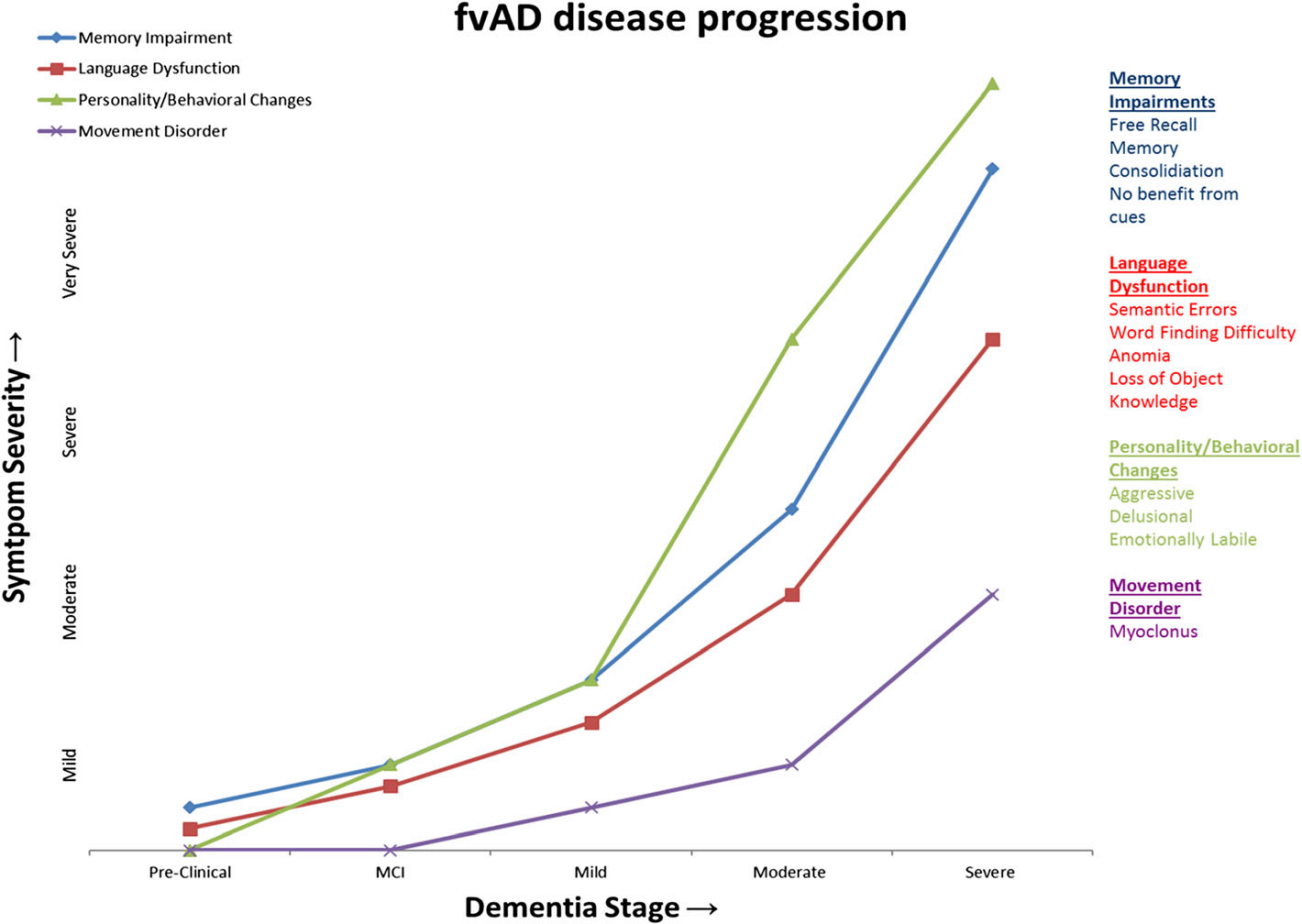
Chronology of symptoms. The diagram illustrates the severity and timing onset of cognitive impairments, motor manifestations, and behavioral changes as they may appear across disease stages in fvAD

Determining the chronology of symptoms is of fundamental importance in the prediction of the underlying pathology of patients with cognitive and behavioral impairments. Patients with bvFTD tend to present with behavioral changes first, which are followed by executive dysfunction and language disturbances (Fig. 3). Conversely, in fvAD memory impairment is the first abnormality in up to 85% of patients (Fig. 4), with the remainder developing memory impairment within 3 years from symptom onset [1, 22, 23]. Our cases highlight the difficulty in determining the sequence of symptoms, and highlight the heterogeneity of and overlap between the fvAD and bvFTD phenotypes. While not apparent in our cases, studies have demonstrated that executive or behavioral dysfunction typically develop after memory deficits in fvAD [1, 22, 23].

### Memory and language dysfunction

*Memory impairment* may be due to disruptions in any of the three stages of memory processing: registration, consolidation, and retrieval. While registration relies upon the integrity of more diffuse attentional and perceptual networks, consolidation hinges on temporal (hippocampal based) networks, and memory retrieval on frontal networks. While bvFTD patients may have mild difficulty with free recall early in the disease, recognition memory is typically intact and thus patients are able to recognize items with cueing, consistent with a retrieval-predominant deficit [24]. Early recall impairment in fvAD is due to the inability to consolidate memories [25]. As memory is not formed, cueing is of no benefit, which was the case in our patients –a missed clue as to the underlying pathology.

*Language impairment* also shows different patterns in bvFTD and fvAD. In bvFTD, language dysfunction is initially absent, unlike other frontotemporal dementia phenotypes, namely progressive nonfluent aphasia and semantic dementia [17]. In our experience, the “social-emotional” aspects of speech, rather than language itself, may be impaired, with inability to understand the subtleties and context of conversations, hence the analogy to a “heartless” Tin Man. Progressive language deterioration may occur late in bvFTD. The fvAD Scarecrow, “who lacks a brain”, may more often present with word-finding difficulties, anomia, and semantic paraphasias. Fluency may also be helpful in differentiating both disorders. While semantic (category) fluency is usually impaired to a greater degree in fvAD, phonemic (letter) fluency is more affected in bvFTD [17]. Interestingly, the use of profanity during verbal fluency testing has been shown to be more suggestive of bvFTD than AD [26]. We hypothesize that the use of pejoratives seen in case #2 was a filler for word-finding difficulty, with over-utilization of an overlearned phrase, rather than an indicator of disinhibition. Case #3, in which the patient had progressive anomia, was more typical of fvAD in retrospect.

On the other hand, executive dysfunction may not be helpful in distinguishing between underlying degenerative processes. Only one study has shown worse executive function in fvAD compared to bvFTD but such difference may have been confounded by the presence of comorbidities such as cerebrovascular disease, sleep apnea, medication side effects, metabolic dysfunction, and inflammatory conditions [21]. The associated cerebrovascular disease in cases #1 and 2 may have contributed to non-specific frontal dysfunction.

### Personality change and behavioral disturbances

*Personality changes* are more common in patients with bvFTD than fvAD, with differences in the nature of the personality change shedding light on the underlying diagnosis [10, 22]. Patients with bvFTD are most likely to exhibit disinhibition, and are often described as socially inappropriate [21]. Moreover, unlike fvAD “Scarecrows”, bvFTD “Tin Men” may be cold, more apathetic and less empathetic, creating tension with caregivers. We hypothesize the mental rigidity of bvFTD prevents shifting viewpoints and fixate on just one. Relatedly, bvFTD patients may exhibit compulsive perseverative thoughts or behaviors, including collecting, hoarding, and hyperphagia, while these compulsive changes are typically absent in fvAD [27].

Recognizing *behavioral changes* may not be obvious, as dementia tends to insidiously exaggerate pre-morbid personality traits. Whereas irritability and depression, as in Case #2, and emotional lability, as in Case #3, may be more common in fvAD, constricted affect and apathy are more typical in bvFTD. The bvFTD “Tin Man” is more emotionally constrained, whereas the fvAD “Scarecrow” is often depressed and may be overburdened by delusions and aggressive behaviors. Each profile creates a distinct caregiver burden in these disorders [22, 27].

One particularly useful clinical scenario to inquire about is meal time. bvFTD patients eating habits may be significantly altered, with hyperphagia and food rituals, and a tendency to seek out and eat one type of food, such as high-carbohydrate meals, with resultant weight gain. This contrasts with the weight loss typical of fvAD patients. Lastly, because of disinhibition and lack of empathy, bvFTD patients have poor table manners, and may engage in eating off tablemates’ plates or uncouthly eating with their hands.

### Motor manifestations

Motor manifestations may yield valuable insights in distinguishing bvFTD from fvAD. As cortical myoclonus is documented in about 50% of patients with fvAD, [28] its presence favors fvAD over bvFTD. The clinician can imagine, for the purpose of mnemonics, the wind jolting fvAD Scarecrow because of spontaneous and stimulus-sensitive cortical myoclonus of the hands and face in contrast with the poorly lubricated bvFTD Tin Man, which instead renders him parkinsonian.

Parkinsonism is indeed present in 20–30% of patients with bvFTD. When prominent, mutations in the microtubule-associated protein tau (*MAPT*) tend to be more common than mutations in *GRN* or *C9orf72* genes [29, 30]. A symmetric tremorless parkinsonism with axial rigidity and supranuclear gaze palsy has been shown to be highly predictive of progressive supranuclear palsy pathology due to *MAPT* or, less commonly, *C9orf72* mutations. On the other hand, a corticobasal syndrome (asymmetric parkinsonism with limb dystonia, apraxia, and cortical sensory loss) is more often due to AD pathology than corticobasal degeneration or other disorders [31]. While parkinsonian features may be present in up to 30% of patients with AD, [32] these appear later in the disease course [32]. The parkinsonian features of Case #2, however, were likely contributed to by Lewy body pathology, which not uncommonly co-occurs with AD. Finally, the coexistence of motor neuron disease strongly suggests pathogenic *C9orf72* mutations [29].

### Ancillary testing

Structural neuroimaging may also provide clues in the assessment of these patients. In fvAD there is greater temporal than frontal atrophy, particularly in the perisylvian area compared to bvFTD [21, 33]. In bvFTD, frontal regions are more affected, particularly the anterior cingulate, orbitofrontal cortex, middle and superior frontal gyrus [21, 33].

Cerebrospinal fluid (CSF) biomarkers studies are invaluable in distinguishing between fvAD from bvFTD and healthy controls [25, 34, 35]. Useful CSF biomarkers include total tau (t-tau), phosphorylated tau (p-tau), and amyloid β-42 (Aβ42). A higher p-tau/Aβ42 ratio (>0.21) is observed in fvAD compared to bvFTD (sensitivity and specificity of about 92%) [25]. In one small series, APOE ε4 carriers were observed in 52% of patients with fvAD compared to 19% with bvFTD and 17% controls [21]. Hence, the presence of APOE ε4 allele makes fvAD more likely than bvFTD.

### Treatment

While no randomized clinical trials have been undertaken in fvAD, acetylcholinesterase inhibitors (donepezil, rivastigmine, and galantamine) have been shown to be effective in classic AD but not in bvFTD [36]. In fact, some bvFTD patients may experience behavioral or cognitive deterioration when exposed to these agents [37]. Likewise memantine, an NMDA receptor antagonist, approved for moderate to severe AD, has shown no benefit in patients with bvFTD [38]. Thus, the different pharmacologic response to treatment in AD versus bvFTD highlights the need to clinically distinguish these disorders. Future clinical trials will require greater sophistication in patient selection not only for symptomatic but also disease-modifying interventions, and will assist efforts in developing reliable serologic and imaging biomarkers of disease trait and progression for each form of frontal dementia.

## Conclusion

Our cases highlight several features, which in hindsight should have supported a diagnostic revision to fvAD from bvFTD: early memory involvement, impaired semantic fluency, paraphasic errors and myoclonus (Table 1). Distinguishing between fvAD and bvFTD may be challenging for the clinician, as evidenced by our three cases whom were initially diagnosed with bvFTD but were found to have primarily AD pathology. Improving the clinical diagnosis of bvFTD and fvAD is important to optimize patient care, improve disease-specific counseling to patients and caregivers, and assist with selective patient recruitment for future studies of disease-modifying interventions. While disease-specific biomarkers remain elusive, larger case series may further refine the Wizard of Oz analogy to help in the clinical distinction between fvAD and bvFTD.

**Table 1:** Clinical features distinguishing fvAD from bvFTD

	Clinical Features supporting fvAD	Clinical Features supporting bvFTD
Memory	Early memory complaints	Late memory complaints
Language	Phonemic and semantic paraphasias	Loss of socioemotional aspects of speech
Fluency	Semantic > phonemic fluency impairment	Phonemic > semantic fluency impairment
Behavioral	Compulsive or perseverative behaviors are uncommon	Collection or hoarding, and ritualistic and disinhibited behaviors (particularly involving food)
Personality Change	Agitation and irritability	Early apathy, disinhibition, loss of empathy
Thought Content	Delusions (theft, infidelity, and paranoid)	Mental rigidity
Body Habitus	Weight loss associated with depression	Weight gain associated with hyperphagia
Movement Disorder	Myoclonus (often mischaracterized as tremor), late parkinsonism	Early parkinsonism
Brain MRI pattern	Symmetric atrophy (temporal > frontal, posterior corpus callosum, and perisylvian)	Symmetric (~*MAPT* mutations) or asymmetric (~*GRN* mutations) frontotemporal atrophy
CSF findings	CSF p-Tau/Aβ42 ratio (>0.21 ng/mL)	CSF progranulin levels (<60 ng/mL)- not validated in clinical practice
Biomarkers	APOE ε4 allele positive	No relation to APOE allele

## Abbreviations

ABC: Amyloid, Braak, and CERAD rating scale
AD: Alzheimer’s disease
bvFTD: Behavioral variant of frontotemporal dementia
C9orf79: Chromosome 9 open reading frame 72
CERAD: Consortium to establish a registry for Alzheimer’s disease
CSF: Cerebral spinal fluid
FAB: Frontal assessment battery
FTLD: Frontotemporal lobar degeneration
fvAD: Frontal variant of Alzheimer’s disease
GRN: Progranulin
MAPT: Microtubule-associated protein tau
MoCA: Montreal cognitive assessment
NIA-AA: National Institute on Aging Alzheimer’s Association
PD: Parkinson’s disease
UPDRS: Unified Parkinson’s disease rating scale
